# Unfolded protein response in Gaucher disease: from human to *Drosophila*

**DOI:** 10.1186/1750-1172-8-140

**Published:** 2013-09-11

**Authors:** Gali Maor, Sigal Rencus-Lazar, Mirella Filocamo, Hermann Steller, Daniel Segal, Mia Horowitz

**Affiliations:** 1 Department of Cell Research and Immunology, Tel Aviv University, Levanon St, Ramat Aviv 69978, Israel; 2 Centro di Diagnostica Genetica e Biochimica delle Malattie Metaboliche, IRCCS G. Gaslini Genoa, Italy; 3 Howard Hughes Medical Institute, Strang Laboratory of Cancer Research, The Rockefeller University, 1230 York Avenue, New York, NY 10021, USA; 4 Department of Molecular Biotechnology and Microbiology, Tel Aviv University, Tel Aviv 69978, Israel; 5 Sagol Interdisciplinary School of Neurosciences, Tel Aviv University, Tel Aviv 69978, Israel

**Keywords:** Gaucher disease, Parkinson’s disease, Glucocerebrosidase, ERAD, UPR

## Abstract

**Background:**

In Gaucher disease (GD), resulting from mutations in the GBA gene, mutant β-glucocerebrosidase (GCase) molecules are recognized as misfolded in the endoplasmic reticulum (ER). They are retrotranslocated to the cytoplasm, where they are ubiquitinated and undergo proteasomal degradation in a process known as the ER Associated Degradation (ERAD). We have shown in the past that the degree of ERAD of mutant GCase correlates with GD severity.

Persistent presence of mutant, misfolded protein molecules in the ER leads to ER stress and evokes the unfolded protein response (UPR).

**Methods:**

We investigated the presence of UPR in several GD models, using molecular and behavioral assays.

**Results:**

Our results show the existence of UPR in skin fibroblasts from GD patients and carriers of GD mutations. We could recapitulate UPR in two different *Drosophila* models for carriers of GD mutations: flies heterozygous for the endogenous mutant GBA orthologs and flies expressing the human N370S or L444P mutant GCase variants. We encountered early death in both fly models, indicating the deleterious effect of mutant GCase during development. The double heterozygous flies, and the transgenic flies, expressing mutant GCase in dopaminergic/serotonergic cells developed locomotion deficit.

**Conclusion:**

Our results strongly suggest that mutant GCase induces the UPR in GD patients as well as in carriers of GD mutations and leads to development of locomotion deficit in flies heterozygous for GD mutations.

## Background

Gaucher disease (GD) is a lysosomal storage disease, caused by mutations in the gene encoding lysosomal acid β-glucocerebrosidase (GCase), designated GBA. As a result, glucosylceramide (GlcCer) is not properly degraded and accumulates primarily in cells of mononuclear phagocyte origin [1,2].

More than 300 mutations were identified in the GBA gene. A large fraction of them are missense mutations, though premature termination, splice site mutations, deletions and recombinant alleles have been recognized as well [3]. There are several abundant mutations. Thus, the N370S mutation [4] is the most prevalent among type 1 GD patients, while the L444P mutation [5] is most common among the neuronopathic types of GD. The majority of patients homozygous for this mutation develop type 3 GD. The 84GG mutation is an insertion of a guanine 84 nucleotides downstream from the first initiator methionine of the GBA mRNA, resulting in premature protein termination [6].

As a lysosomal enzyme, GCase is synthesized on endoplasmic reticulum (ER) bound polyribosomes [7]. Upon its entry into the ER, it undergoes N-linked glycosylation on four asparagines, after which it is subject to ER quality control (ERQC). When correctly folded it shuttles to the Golgi compartment for further modifications on the N-glycans and finally it traffics to the lysosomes. Mutant GCase variants are recognized as misfolded proteins and undergo various degrees of ER associated degradation (ERAD). The accumulation of misfolded molecules in the ER, activate signaling events known as the unfolded protein response (UPR) [8]. UPR monitors the conditions in the ER, by sensing insufficiency in protein folding capacity and translates this information into gene expression [9]. The ER membrane harbors three ER stress sensors: The type 1 transmembrane protein kinase endoribonuclease IRE1, the type 1 protein kinase PERK, and the activating transcription factor 6 (ATF6). These three branches operate simultaneously and use unique mechanisms of signal transductions. The three UPR transducers are constitutively expressed in metazoan cells [10], and are maintained in an inactive state through interaction with the ER protein chaperone BiP. Accumulated unfolded protein(s) binds and sequesters BiP, thus promoting BiP dissociation from PERK, IRE1 and ATF6. Dissociation of BiP from the three stress sensors allows their modification and signal transduction, which results in a response to the accumulation of misfolded proteins [11]. Thus, IRE1 undergoes dimerization and phosphorylation and participates in a cytoplasmic complex, which splices the transcription factor X-box binding protein 1 (Xbp1). Upon its splicing the Xbp1 mRNA is translated into a protein that translocates into the nucleus and turns on UPR related genes [9,12,13]. PERK is a kinase that undergoes dimerization and autophosphorylation and mediates phosphorylation of the eukaryotic translation initiation factor 2α (eIF2α). Phosphorylated eIF2α attenuates general protein translation in the cells [9,11,14]. ATF6, the third component among the UPR sensors, shuttles to the Golgi, where it is sequentially cleaved by proteases. Its cleaved N-terminal cytosolic fragment enters the nucleus where it serves as a transcription factor of UPR upregulated genes, including the induction of the proapoptotic bZIP transcription factor CCAAT/enhancer-binding protein homologous protein (CHOP) [15], which is essential for cell cycle arrest and the apoptotic response to chronic ER stress [9,11,13,14,16]. Manifestation of UPR in GD derived cells has already been noted in cell lines that originated from GD patients, homozygous for the N370S or the L444P mutations [17,18]. Yet, accumulation of glucosylceramide (GlcCer) per se, induced by conduritol-β-epoxide (CBE), did not result in UPR [19]. Likewise, in the absence of mutant GCase there was no UPR [19], underscoring the importance of mutant GCase in the activation of ERAD and UPR.

In this study we tested whether UPR is activated in GD. Our results show the occurrence of UPR in GD derived skin fibroblasts and in carriers of GD mutations, both in humans and *Drosophila*. In heterozygous flies, and flies expressing the human N370S or L444P mutant GCase variants there were significant developmental defects. Locomotion deficit was evident in aging flies, reminiscent of Parkinson disease (PD).

## Materials and methods

### Materials

The following primary antibodies were used in this study: rabbit polyclonal anti-GRP78 antibodies (Cell Signaling Technology, Beverly, MA, USA), mouse monoclonal anti-CHOP antibody (Cell Signaling Technology, Beverly, MA, USA), rabbit polyclonal anti-phospho-eIF2α (Ser51) antibodies, rabbit polyclonal anti-eIF2α antibodies (from cell signaling Technology, Beverly, MA, USA), mouse monoclonal anti-actin antibody (Sigma-Aldrich, Israel), rabbit polyclonal anti human GCase antibodies (Sigma-Aldrich, Israel) and mouse monoclonal anti-tubulin antibody (Sigma-Aldrich, Israel).

Secondary antibodies used were: horseradish peroxidase-conjugated goat anti-mouse antibodies and Horseradish peroxidase-conjugated goat anti-rabbit antibodies (both from Jackson Immuno Research Laboratories, West Grove, PA, USA). Leupeptin, phenylmethylsulfonyl fluoride (PMSF) and aprotinin were from Sigma–Aldrich (Rehovot, Israel). Absolute Blue qPCR SYBR Green ROX Mix was from Thermo Scientific (Logan, UT, USA).

### Cell lines

Human primary skin fibroblasts were provided by two publically available, sources: by “Cell Line and DNA Biobank from Patients Affected by Genetic Diseases” (G. Gaslini Institute), Telethon Genetic Biobank for GD skin fibroblasts or by Prof. R. O. Brady, NIH. The patients signed an informed consent. Work with the cell lines was in accordance with the institutional guidelines of Tel Aviv University. Identifiable clinical and personal data from the patients were not available for this study. Cells were grown in DMEM supplemented with 20% FBS (Biological Industries, Beit Haemek, Israel). All cells were grown at 37°C in the presence of 5% CO2.

### Fly strains

Canton-S flies (WT) served as a wild-type control. Strains were maintained on cornmeal-molasses medium at 25°C. Strains harboring a minos transposable element in CG31414 [Mi{ET1}CG31414] or in CG31148 [Mi{ET1}CG31148] were from the Bloomington Stock Center (#23602 and #23435, respectively). *Da-Gal4* and *Ddc-Gal4* driver lines were obtained from Bloomington Stock Center. Transgenic flies, harboring pUASTmycHisGCase, pUASTmycHisN370SGCase or pUASTmycHisL444PGCase on the second chromosome, were established by BestGene (Chino Hills, CA, USA).

### Methods

#### Construction of plasmids

An *Xba*I-*Sap*I fragment, isolated from pcDNA4 (Invitrogen Life Technologies Co., Carlsbad, CA, USA) was subcloned between *Xba*I and *Sap*I restriction sites of pUAST, to create pUASTmycHis. *Eco*RI-*Xho*I fragments, containing either the normal or the N370S or the L444P mutant human GCase cDNAs, isolated from the plasmids MycHis WT GCase, MycHis N370S GCase or MycHis L444P GCase [20], respectively, were subcloned in pUASTmycHis, cleaved with the same restriction enzymes, to create pUASTmycHisGCase, pUASTmycHisN370SGCase or pUASTmycHisL444PGCase, respectively.

#### RNA preparation

Total RNA was isolated using EZ-RNA kit (Biological Industries, Beit Haemek, Israel), according to the manufacturer’s instructions. For RNA extraction from flies, adult flies were frozen in liquid nitrogen and then homogenized in TRI Reagent solution (MRC, Cincinnati, Ohio, USA). The extraction was performed according to the manufacturer’s recommendations.

#### RT PCR

Two μg of RNA were reverse transcribed with M-MLV reverse transcriptase (Promega Corporation, CA, USA), using oligo dT primer in a total volume of 20 μl, at 42°C for 60 minutes. Reactions were stopped by incubation at 70°C for 15 minutes. One-two microliters of the resulting cDNA were amplified by PCR or by quantitative real time PCR.

#### PCR

PCR was executed in 25 μl containing 0.4 mM dNTPs, 10 ρM of each primer, 1 unit of *Taq* polymerase (Takara, Shiga, Japan) and 10× *Taq* buffer (10 mM Tris HCL pH 8.3, 50 mM KCl and 1.5 mM MgCl_2_). Thirty cycles of 94°C (1 minute), 58°C (1 minute) and 72°C (1 minute) were performed, following by 10 minutes at 72°C for final extension. PCR reactions were carried out in an Eppendorff Master-cycler EP Gradient S (Eppendorf, Hamburg, Germany). PCR products were separated by agarose gel electrophoresis (1–1.5%) and visualized with 0.1% ethidium bromide. Sequence of the primers used appears in Table 1.

**Table 1:** Primers used in this study

	
Human-GAPDH-RT-F	5′-CTCCTCCTGTTCGACAGTCA-3′
Human-GAPDH-RT-R	5′-GTTGACTCCGACCTTCACCT-3′
Human-CHOP-RT-F	5′-AGCGACAGAGCCAAAATCAG-3′
Human-CHOP-RT-R	5′-TCTGCTTTCAGGTGTGGTGA-3′
Human-GRP78-RT-F	5′-CATCAAGTTCTTGCCGTTCA-3′
Human-GRP78-RT-R	5′-ATGTCTTTGTTTGCCCACCT-3′
Human-GAPDH-F	5′-CCATCAATGACCCCTTCATTGACC-3′
Human-GAPDH-R	5′-CTCAYGGYYCACACCCATGAC-3′
Human-s-XBP1-F	5′-TCTGCTGAGTCCGCAGCAG-3′
Human-s-XBP1-R	5′-GAAAAGGGAGGCTGGTAAGGAAC-3′
*Drosophila*-Hsc-70-3-RT-F	5′-GCTGGTGTTATTGCCGGTCTGC-3′
*Drosophila*-Hsc-70-3-RT-R	5′-GATGCCTCGGGATGGTTCCTTGC-3′
*Drosophila*-s-Xbp1-RT-F	5′-CCGAACTGAAGCAGCAACAGC-3′
*Drosophila*-s-Xbp1-RT-R	5′-GTATACCCTGCGGCAGATCC-3′
*Drosophila*-RP49-RT-F	5′-TAAGAAGCGCACAAAGCACT-3′
*Drosophila*-RP49-RT-R	5′-GGGCATCAGATATTGTCCCT-3′

The table contains the sequence of all the primers used in this work. *RT* real time, *R* reverse, *F* forward.

#### Quantitative real time PCR

One μl of cDNA was used for quantitative real time PCR. PCR was performed using “power SYBR green QPCR mix reagent kit” (Applied Biosystems, Foster City, CA, USA) by Rotor-Gene 6000 (Qiagen, Valencia, CA, USA). The reaction mixture contained 50% QPCR mix, 300 nM of forward primer and 300 nM of reverse primer, in a final volume of 10 μl. Thermal cycling conditions were 95°C (10 minutes), and 40 cycles of 95°C (10 seconds) 60°C (20 seconds) and 72°C (20 seconds). Relative gene expression was determined by Ct value. Human cDNA was amplified with primers specific for human BiP (Human-GRP78-RT-F and Human-GRP78-RT-R, Table 1) or human CHOP (Human-CHOP-RT-F and Human-CHOP-RT-R, Table 1). GAPDH was used as a normalizing control for human genes (amplified with primers: Human-GAPDH-RT-F and Human-GAPDH-RT-R, Table 1). Amplification of *Drosophila* genes was conducted with primers specific for *Drosophila* Hsc-70-3 (*Drosophila*-Hsc-70-3-RT-F and *Drosophila*-Hsc-70-3-RT-R, Table 1), or for the spliced form of *Drosophila* Xbp1 (*Drosophila*-s-Xbp1-RT-F and *Drosophila*-s-Xbp1-RT-R, Table 1). RP49 was used as a normalizing control (amplified with primers: *Drosophila* RP49-RT-F and *Drosophila* RP49-RT-R, Table 1).

#### Detection of spliced Xbp1 mRNA processing

Human spliced Xbp1 was amplified from cDNA using the primers: Human s-Xbp1 F and Human s-Xbp1-R (Table 1). GAPDH was used as a normalizing control (amplified with primers: Human-GAPDH-F and Human-GAPDH-R, Table 1). To amplify *Drosophila* spliced Xbp1 the primers: *Drosophila* s-Xbp1-RT-F and *Drosophila* s-Xbp1-RT-R (see Table 1) were used, with *Drosophila* RP49 as a normalizing control. The forward primer could anneal only to the spliced form of Xbp1 mRNA.

### SDS-PAGE and western blotting

#### Cultured cells

Cell monolayers were washed three times with ice-cold phosphate-buffered saline (PBS) and lysed at 4°C in lysis buffer (10 mM HEPES pH 8.0, 100 mM NaCl, 1 mM MgCl2 and 1% Triton X-100) containing 10 μg/ml aprotinin, 0.1 mM phenylmethylsulfonyl fluoride (PMSF) and 10 μg/ml leupeptin. Lysates were incubated on ice for 30 minutes and centrifuged at 10,000 g for 15 minutes at 4°C.

#### Flies

For each preparation, 10 flies were homogenized in RIPA lysis buffer (50 mM Tris/HCL, 150 mM NaCl, 1 mM EDTA, 1% TritonX-100, 1% sodiumdeoxycholate, 0.1% SDS) containing protease inhibitors (10 μg/ml leupeptin, 10 μg/ml aprotinin and 0.1 mM PMSF- all from Sigma-Aldrich, Israel). Samples containing the same amount of protein were electrophoresed through 10% SDS–PAGE and electroblotted onto a nitrocellulose membrane (Schleicher and Schuell BioScience, Keene, NH, USA). Further treatment of membranes and ECL detection was as described elsewhere [21].

### Enzymatic activity

Confluent primary skin fibroblasts were washed twice with ice-cold PBS and collected with a rubber policeman in 150 μl sterile water. Cell lysates, containing 40 μg of protein, were assayed for GCase activity in 0.2 ml of 100 mM potassium phosphate buffer, pH 4.5, containing 0.15% Triton X-100 (Sigma-Aldrich, Israel) and 0.125% taurocholate (Calbiochem, La Jolla, CA, USA) in the presence of 1.5 mM 4-methyl-umbeliferyl-glucopyranoside (MUG) (Genzyme Corporation. Boston, MA, USA) for 1 h at 37°C. The reaction was stopped by the addition of 0.5 ml of stop solution (0.1 M glycine, 0.1 M NaOH, pH 10) and the amount of 4-methyl-umbeliferone (4-MU) was quantified using Perkin Elmer Luminescence Spectrometer LS 50 (excitation wavelength: 340 nm; emission: 448 nm) [22].

### Endonuclease-H (endo-H) treatment

Samples of cell lysates, containing 100 μg of total protein, were subjected to an overnight incubation with endo-H (New England Biolabs, Beverly, MA, USA), according to the manufacturer’s instructions. They were electrophoresed through 10% SDS-PAGE and the corresponding blot was interacted with anti GCase and anti actin antibodies. Total GCase amount was divided by that of actin at the same lane and normalized to WT GCase, which was considered 100. To determine the endo-H resistant fraction, the intensity of GCase resistant fraction was divided by the intensity of the entire amount of GCase in the same lane. GDPV (GD Predictive Value) was determined as described elsewhere [23] (GCase amount X GCase resistant fraction: 100).

### Climbing assay of flies

Vials, each containing 10 male flies, were tapped gently on the table and left standing for 15 seconds. The number of flies that climbed at least five cm was recorded. The experiment was repeated 10 times.

### Blot quantitation

The blots were scanned using Image Scan scanner (Amersham Pharmacia Biotech, Buckinghamshire, England), and the intensity of each band was measured by the Image Master 1DPrime densitometer (Amersham Pharmacia Biotech, Buckinghamshire, England) and GelQuant (BiochemLabSolutions).

### Statistics

All the results were statistically analyzed using the student *t*-test.

## Results

### Activation of UPR in GD derived fibroblasts

Mutant GCase is recognized as misfolded in the ER. After several unsuccessful attempts to refold it, it undergoes ERAD [21]. The level of ERAD correlates with GD severity, since it determines the amount of mutant enzyme that reaches the lysosomes and degrades the substrate there, depending on its residual activity. Moreover, skin fibroblasts that derived from GD patients homozygous for the N370S or the L444P mutations exhibited UPR [17,24]. We, therefore, decided to extend the study and tested whether UPR is activated in additional GD derived cells. The UPR induces increased transcription of the molecular chaperone BiP and the transcription factor CHOP [25]. In addition, the UPR induces splicing of the Xbp1 transcript and phosphorylation of eIF2α [25].

**Figure 1:**
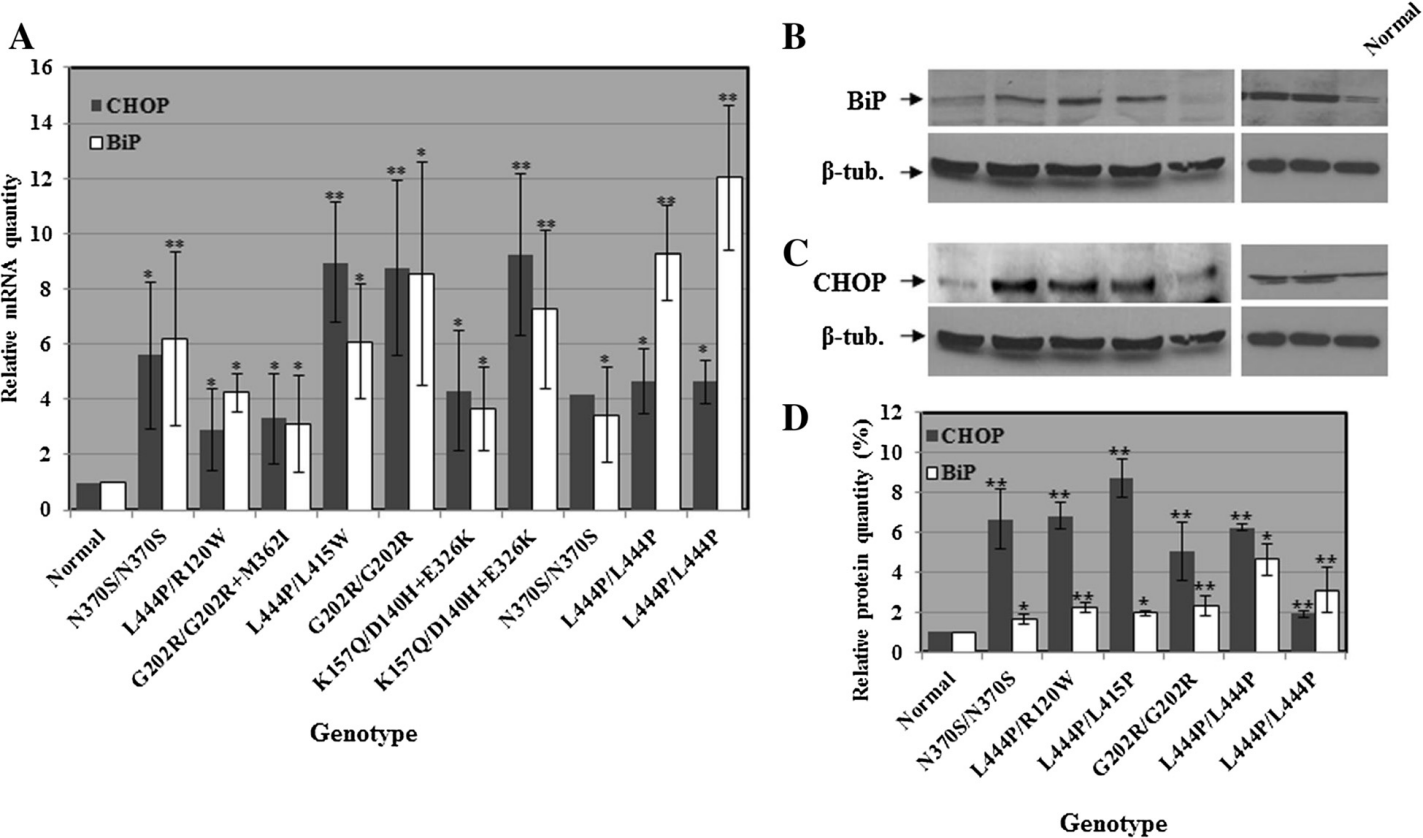
**Elevation in CHOP and BiP levels in GD derived cells. A.** RNA was isolated from different GD derived skin fibroblasts, and the corresponding cDNA was used for quantitative RT-PCR with primers specific for human BiP or CHOP. GAPDH was used as a normalizing control. Two different normal cell lines were used as control. Dark box: CHOP; Light box: BiP. **B, C.** Protein lysates were prepared from different GD derived skin fibroblasts and subjected to western blotting. The corresponding blots were interacted with anti BiP **(B)** and anti CHOP **(C)** antibodies. As a loading control, the blots were interacted with anti-tubulin antibody. For each protein there are two blots, each with a normal control. This is due to the fact that cell lysates were prepared at different times, depending on the growth rate of the cell lines and, therefore, ran on different gels. The genotypes for **B** and **C** are shown in **D**. **D.** The blots were quantified as explained. The amount of BiP and CHOP was divided by that of tubulin in the same lane, and the values obtained for normal cells were considered 1. The results are the mean (minus plus standard error) of three different experiments. Significance: * < 0.05; ** < 0.01.

Based on the above, we first examined the activation of UPR in GD by testing mRNA levels of BiP and CHOP in skin fibroblasts obtained from GD patients. To do so, we used the quantitative RT-PCR approach, using normal fibroblasts as control. Our results, (Figure 1A), showed a significant increase in BiP and CHOP mRNA levels in GD derived fibroblasts, compared to normal cells. A concomitant increase was detected in the protein levels of BiP and CHOP (Figure 1B-D).

In GD, there is accumulation of the GCase substrate GlcCer, along with the presence of mutant GCase, which undergoes ERAD. In order to test possible contribution of substrate accumulation to UPR, we induced substrate accumulation using the non-competitive inhibitor of GCase, CBE, for 10 days, as has done by Farfel et al. [19]. It has already been shown in the past that CBE treatment leads to substrate accumulation in skin fibroblasts [26]. Treatment of normal skin fibroblasts with 200 μM CBE, which completely abolished GCase activity, (Additional file 1: Figure S1A) did not induce elevation in mRNA levels of either BiP or CHOP (Additional file 1: Figure S1B). Thus, substrate accumulation in GD derived fibroblasts does not activate the UPR.

### Splicing of Xbp1 as a UPR marker in GD derived fibroblasts

**Figure 2:**
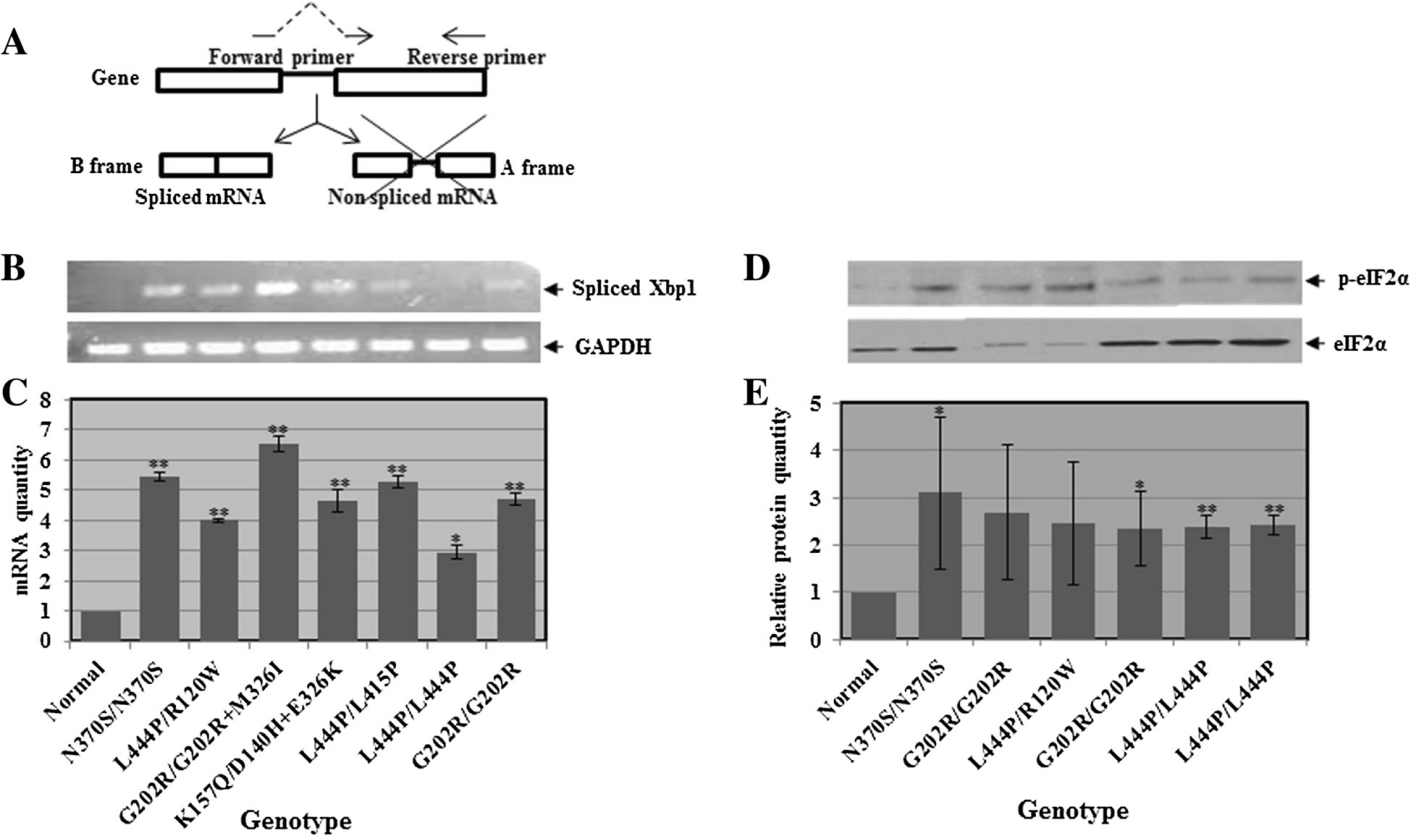
**Elevation in Xbp1 splicing and eIF2α phosphorylation in GD derived cells. A.** Scheme showing the two Xbp1 RNA variants, the spliced and the non-spliced forms. **B.** RNA was isolated from different GD derived skin fibroblasts, and the cDNA prepared from it was used for RT-PCR, with primers specific for the spliced form of human Xbp1. GAPDH was used as a normalizing control. **C.** The results (three different experiments) were quantified and the amount of Xbp1 was divided by that of GAPDH in the same lane. The values obtained for normal cells were considered 1. **D.** Protein lysates were prepared from different GD derived skin fibroblasts and subjected to western blotting. The corresponding blots were interacted with anti phosphorylated eIF2α (p-eIF2α) and as a loading control, with anti eIF2α antibodies. **E.** p-eIF2α amount was divided by that of eIF2α in the same lane, and the values obtained for normal cells were considered 1. The results are the mean (minus plus standard error) of three different experiments. Significance: * < 0.05; ** < 0.01. The genotypes for **B** and **D** are shown in **C** and **E**, respectively.

Splicing of Xbp1 is a central hinge of the IRE1 pathway [25,27], which is another branch activated in the UPR. The Xbp1 mRNA contains two overlapping reading frames, A and B. Under normal conditions, only A frame is transcribed, producing an unspliced version of Xbp1 with no protein product (see Figure 2A) [28]. Upon UPR activation, IRE1 dimerizes and participates in cytoplasmic splicing of Xbp1, thus, removing a 26 bp intron from the Xbp1 mRNA. The spliced Xbp1 mRNA (frame B) encodes a transcription factor that binds to the UPRE or ERSE consensus sequences of promoters of UPR target genes, thus leading to their transcription [28]. Expression of spliced Xbp1 in GD derived skin fibroblasts was tested (see Figure 2B). Our results, presented in Figure 2B, showed that in GD derived fibroblasts the spliced Xbp1 product was significantly elevated, in comparison to normal fibroblasts.

### Elevation in phosphorylation of eIF2α in GD derived fibroblasts

Dissociation of PERK from BiP leads to its dimerization and autophosphorylation, and mediates phosphorylation of the eukaryotic translation initiation factor 2α (eIF2α). eIF2α is a subunit of eIF2, a heterotrimeric GTPase, required to bring the initiator methionyl-tRNA to the 40S ribosomal subunit for AUG initiation codon selection. Phosphorylation of eIF2α inhibits the GDP/GTP exchange reaction on eIF2, thus preventing eIF2 recycling and its initial step of protein synthesis [9]. Phosphorylated eIF2α attenuates general protein translation in cells [9,11,14]. We therefore tested possible changes in the levels of phosphorylation of eIF2α, using western blotting and interaction with anti-phosphorylated eIF2α antibodies. Our results, presented in Figure 2D, E, showed that eIF2α phosphorylation was increased in GD derived fibroblasts in comparison to normal fibroblasts.

### Activation of UPR in carriers of GD mutations

**Figure 3:**
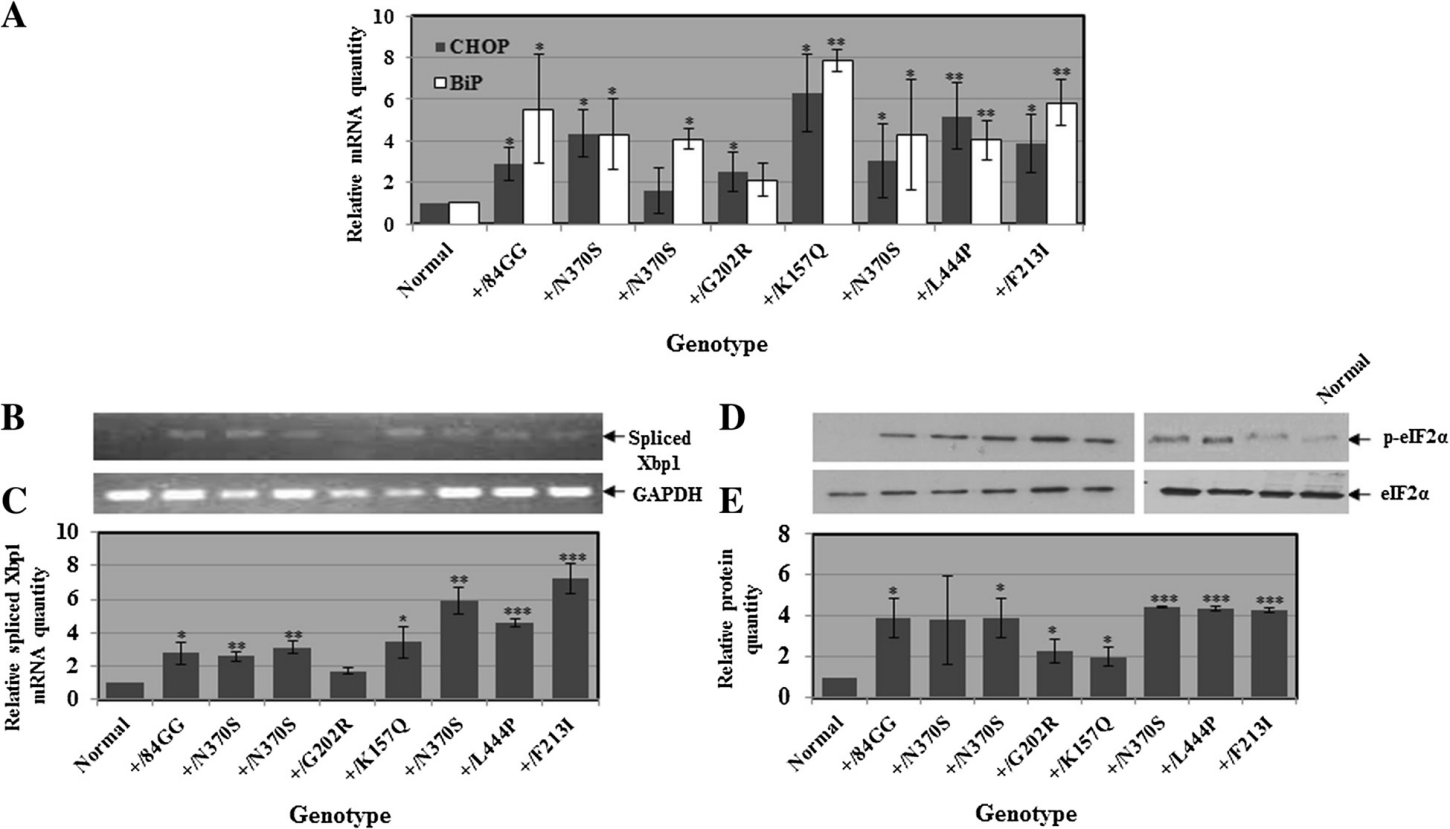
**Activation of UPR in carriers of GD mutations. A.** RNA was isolated from skin fibroblasts that originated from carriers of GD mutations, and the corresponding cDNA was used for quantitative RT-PCR with primers specific for human BiP or CHOP. GAPDH was used as a normalizing control. **B.** cDNA, prepared as in **(A)**, was subjected to RT-PCR, with primers specific for the spliced form of human Xbp1. GAPDH was used as a normalizing control. **C.** The results (three different experiments) were quantified as explained in the legend to Figure 2 and the values obtained for normal cells were considered 1. **D.** Protein lysates, prepared from the above-mentioned cells, were subjected to western blotting and interaction with anti phosphorylated eIF2α antibodies (p-eIF2α). As a loading control, the blots were interacted with anti eIF2α antibodies. **E.** The results (three different experiments) were quantified as explained in the legend to Figure 3B, and the values obtained for normal cells were considered 1. There are two blots, each with a normal control. This is due to the fact that cell lysates were prepared at different times, depending on the growth rate of the cell lines and, therefore, ran on different gels. Significance: * < 0.05; ** < 0.01; *** < 0.005. The genotypes for **B** and **D** are shown in **C** and **E**, respectively.

Our results strongly suggested that UPR results from the presence of mutant GCase in the cells, since substrate accumulation by itself did not lead to activation of the UPR machinery. To confirm our observations we decided to investigate the occurrence of UPR in cells derived from carriers of different GD mutations. To this end we tested elevation in BiP and CHOP mRNA, in Xbp1 splicing and in phosphorylation of eIF2α in cells that derived from carriers of several GD mutations. Our results, presented in Figure 3, showed a significant elevation in the amount of BiP and CHOP mRNAs as well as in Xbp1 splicing in carriers of GD mutations. Likewise, phosphorylation of eIF2α increased significantly in cells that originated from carriers in comparison to normal cells. Interestingly, we observed activation of UPR also in cells carrying the 84GG mutation, strongly suggesting that existence of a mutant GBA mRNA evokes UPR.

### Activation of UPR in *Drosophila* GD-like carriers

**Figure 4:**
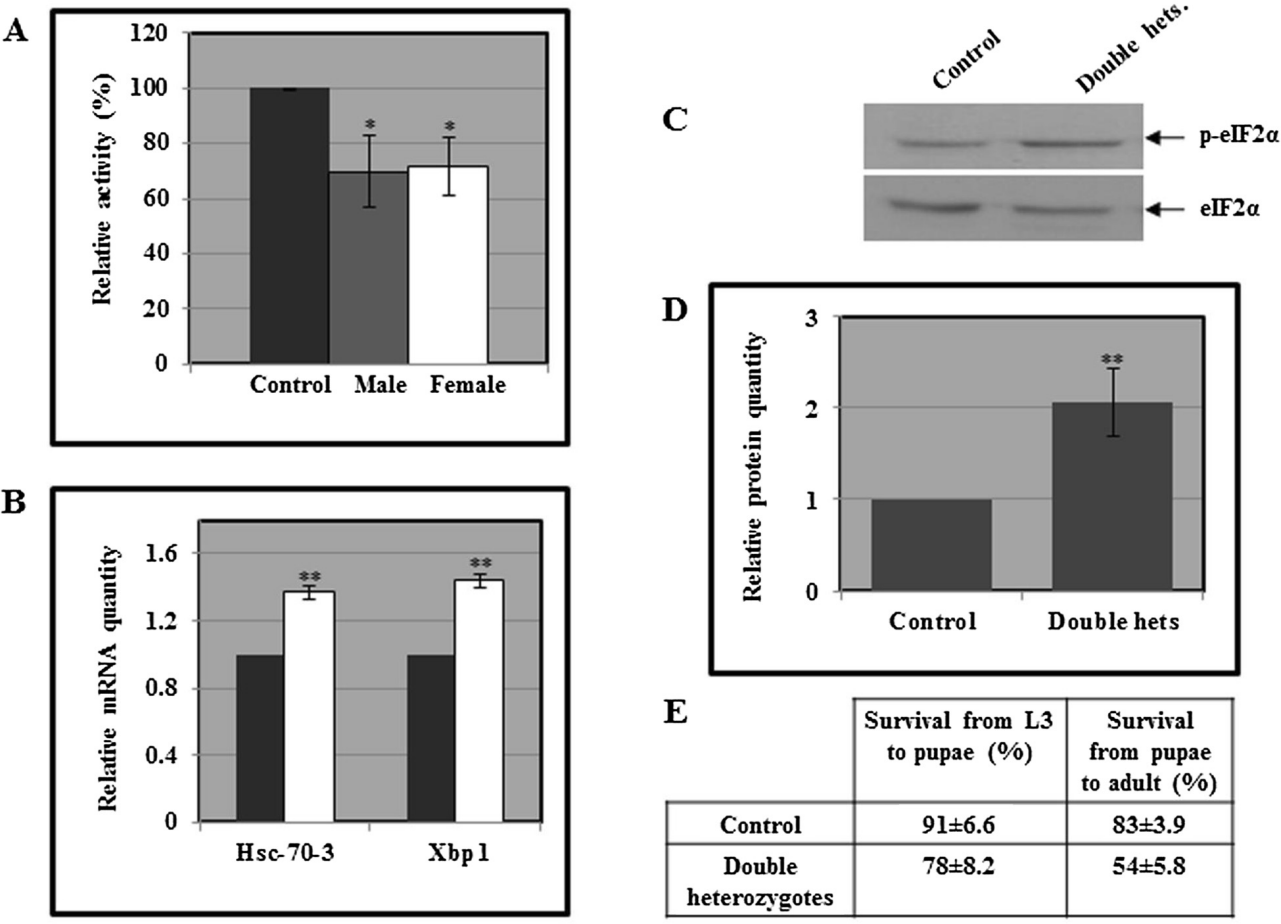
**UPR activation in *Drosophila *carriers of mutant GBA orthologs. A.** GCase activity was tested in protein lysates prepared from either adult wild type (Canton S, WT) flies or from males and females of double heterozygous flies (Mi{ET1}CG31148,CG31414/CG31148,Mi{ET1}CG31414) using 4-MUG as a substrate. Presented are results of three experiments. **B.** RNA was isolated from double heterozygous flies, and the cDNA prepared from it was subjected to quantitative RT-PCR with primers specific for *Drosophila* Hsc-70-3 or for the spliced form of *Drosophila* Xbp1. The results (three different experiments) were quantified as explained in the legend to Figure 1A, and the values obtained for WT flies were considered 1. RP49 was used as a normalizing control. Dark box: WT flies; light box: double heterozygous flies. **C.** Protein lysates from double heterozygous flies (Double hets.) were subjected to western blotting and interaction with anti phosphorylated eIF2α antibodies (p-eIF2α). **D.** The results (three different experiments) were quantified as explained in the legend to Figure 2E and the values obtained for WT flies were considered 1. Significance: * < 0.05; ** < 0.01. **E.** The number of larvae, (L3,) that survived to the pupal stage, and the number of adults that eclosed was counted. The number of larvae taken for each experiment was 90.

There are two GBA homologs in *Drosophila*, designated CG31414 and CG31148, both encoding proteins showing ~31% identity and ~49% similarity to the human GCase. Two *Drosophila* strains are available, each carrying a minos transposable element insertion in one of the fly GBA orthologs. Insertion of minos in the CG31414 gene leads to translation of truncated GCase, lacking 129 C-terminal amino acids (out of the 448 amino acids of the predicted normal fly GCase protein). The minos insertion in CG31148 leads to a truncated protein lacking 34 C-terminal amino acids. Due to their close proximity on chromosome 3, simple genetic manipulations cannot be employed to create a chromosome with both mutated genes. However, double heterozygous fly is an authentic model for the GD carrier state in human. We generated double heterozygous flies, which exhibited ~30% decrease in GCase activity, as expected (Figure 4A). We then tested possible activation of UPR in these flies. The fly does not contain a CHOP homolog in its genome and UPR activation is measured by elevation in mRNA of the BiP ortholog heat-shock cognate 70–3 (Hsc-70-3 gene), in level of Xbp1 splicing and in the level of phosphorylated eIF2α. We observed a significant elevation in the level of Hsc-70-3 mRNA and Xbp1 splicing in the double heterozygotes, as well as in phosphorylation of eIF2α (Figure 4), compared to WT flies. These results clearly point to existence of UPR in carriers of mutations in the GBA orthologs of *Drosophila*. A significant defect in development from larva to pupa and from pupa to adult was found in the double heterozygous animals, in comparison to WT *Drosophila* (Figure 4E). This highlights the significance of normal GCase during development and the deleterious effect of mutant GCase.

**Figure 5:**
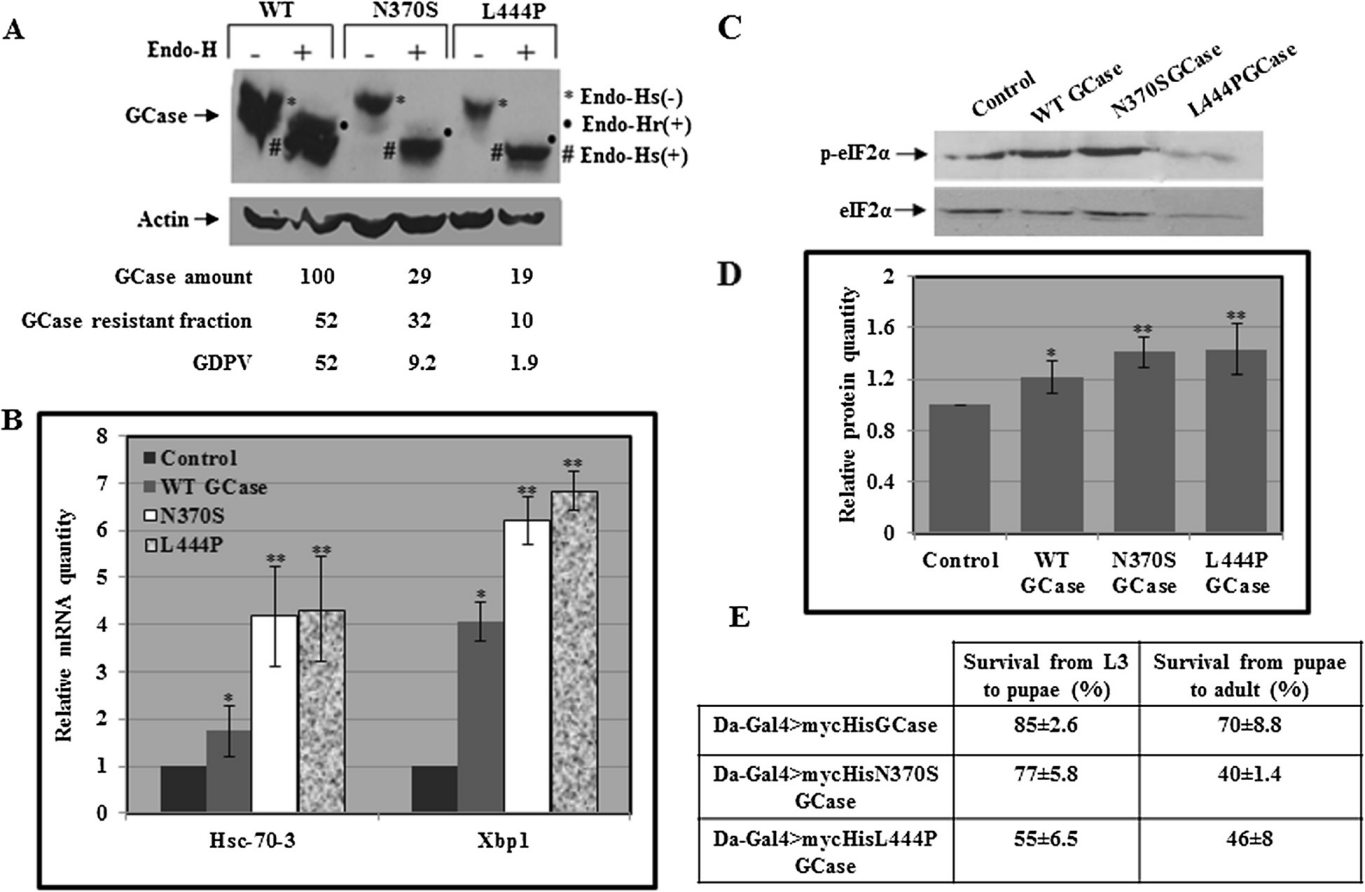
**Activation of UPR in *Drosophila *expressing the human N370S or L444P mutant variants. A.** Protein lysates were prepared from 10 flies expressing the human UAS-mycHisWTGCase, human UAS-mycHisN370SGCase, or human UAS-mycHisL444PGCase, driven by *Da*^*Gal4*^. Samples, containing 100 μg of protein, were subjected to overnight endo-H digestion, after which they were electrophoresed through SDS-PAGE and the corresponding blot was interacted with anti GCase and anti actin antibodies. Total GCase amount was divided by that of actin at the same lane and normalized to WT GCase, which was considered 100. The endo-H sensitive fraction before and after endo-H treatment, and the endo-H resistant fraction were labeled for convenience, as follows: endo-H sensitive before treatment: * [Endo-Hs(−)]; endo-H sensitive after treatment: # [Endo-Hs(+)]; endo-H resistant: • [Endo-Hr(+)]. **B.** RNA was isolated from the above-mentioned flies, and the cDNA prepared from it was subjected to quantitative RT-PCR with the appropriate primers. RP49 was used as a normalizing control. The results (three different experiments) were quantified as explained in the legend to Figure 3B and the values obtained for flies expressing normal human GCase were considered 1. Significance: * < 0.05; ** < 0.01. **C.** Protein lysates, prepared from the above–mentioned flies, were subjected to western blotting and interaction with anti phosphorylated eIF2α antibodies (p-eIF2α). As a loading control, the blot was interacted with anti eIF2α antibodies. **D.** The results (three different experiments) were quantified as explained in the legend to Figure 3B and the values obtained for wild type flies were considered 1. **E.** The number of larvae, (L3), that survived to the pupal stage, and the number of pupae that eclosed was counted. The number of larvae taken for each experiment was 90.

We also ectopically expressed the N370S or the L444P human mutant GCase variants in the fly, using the Gal4/UAS system. In this system the yeast Gal4 transcription factor, expressed from a tissue specific *Drosophila* promoter, is used to express a specific transgene (in our case, the human normal or mutant mycHis tagged GCase cDNAs) coupled to the yeast UAS. Thus, expression of the transgene depends on the specificity of the promoter used for the expression of the Gal4 transcription factor. We used *daughterless-Gal4*, which drives ubiquitous expression of the transgene. We verified expression of the transgene using western blotting and interaction with anti myc antibody (Figure 5A). The results showed expression of the normal human as well as the mutant proteins in the transgenic flies. In comparison to the normal human protein expressed in the fly, the N370S or the L444P mutant variants presented higher endo-H sensitivity (Figure 5A) [21,29,30], illustrating the existence of ERAD of mutant human GCase in the flies. The fraction of lysosomal N370S human GCase, (endo-H resistant fraction, labeled by black circles in Figure 5A) was higher than that of L444P human GCase, as expected for these two mutations and shown for endogenous human N370S and L444P mutant GCase variants [21,29]. Furthermore, the results (Figure 5B-D) demonstrated higher activation of the UPR in flies expressing the mutant variants than those expressing the WT human GCase. UPR was measured by increase in the mRNA levels of Hsc-70-3, in the mRNA levels of spliced Xbp1 and in the levels of phosphorylated eIF2α.

A significant defect in development from larva to pupa and from pupa to adult was found in animals expressing the human mutant GCase variants, in comparison to animals expressing the WT human GCase (Figure 5E), similar to the double heterozygous flies. This highlights the fact that overexpression of human mutant GCase in the fly leads to deleterious effects, regardless of the presence of endogenous WT GCase.

### Locomotor dysfunction in flies expressing human mutant GCase variant

**Figure 6:**
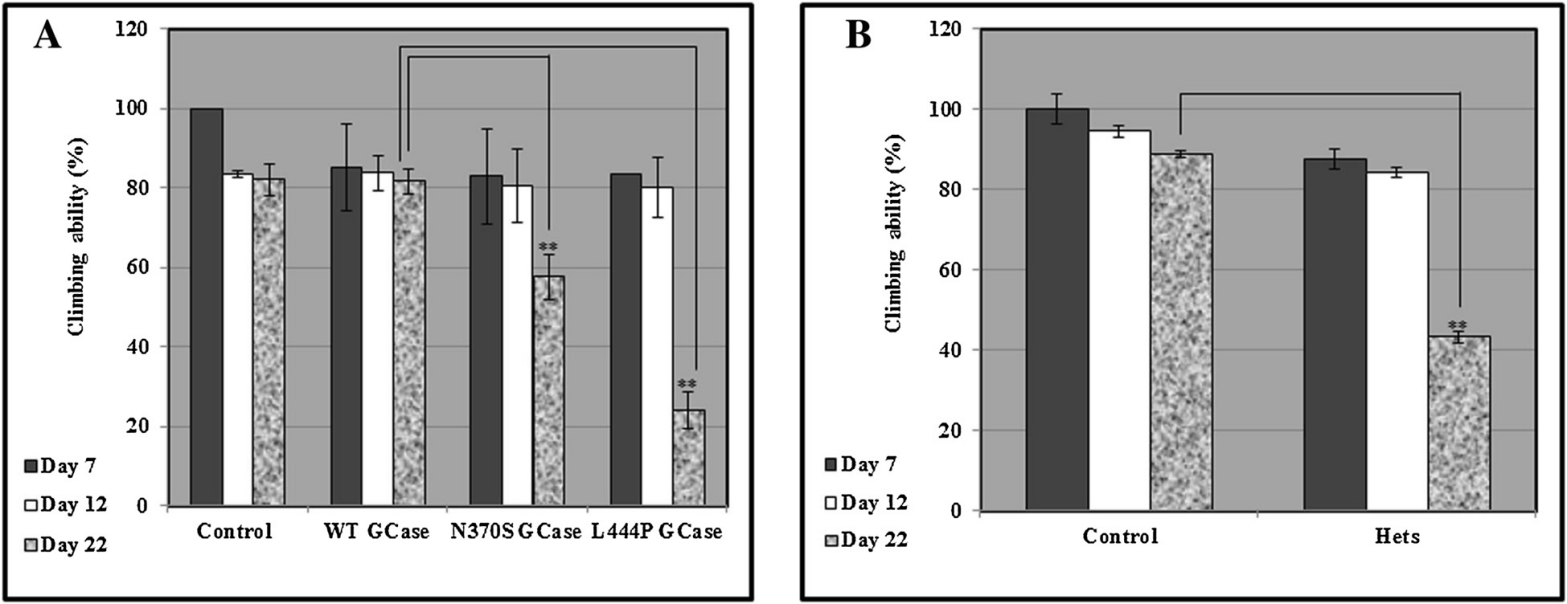
**Locomotion test in flies. A.** Five vials, each containing flies expressing the human UAS-mycHiswtGCase, human UAS-mycHisN370SGCase, or human UAS-mycHisL444P GCase, driven by the *Ddc*Gal4 driver, were analyzed for locomotion behavior. **B.** The same experiment as described in **A** was performed using the WT and double heterozygous flies.

An association has been found between GD patients and carriers of GD mutations and PD. Thus, GD patients and carriers of GD mutations are prone to develop PD [31-55]. We used the *Ddc-Gal4* driver in order to drive specific expression of the human N370S and the L444P GCase mutants in the dopaminergic/serotonergic neurons of the fly. The locomotion (climbing) assay, commonly used for assaying behavior of flies expressing familial Parkinson related proteins in their brain [56], was used to assess the neural dysfunction caused to the flies due to expression of mutant GCase in the nervous system. We monitored the climbing ability of the flies at the age of 7, 12 and 22 days. Measurements, conducted at the age of 22 days, revealed significant locomotion dysfunction in both fly models for carriers of GD mutations, namely, flies double heterozygotes in their two GBA orthologs and flies transgenic for the N370S or the L444P mutant human GCase variants (Figure 6).

## Discussion

In this work we show that persistent presence of mutant GCase activates the UPR, in humans and in *Drosophila*.

Several previous publications have already noticed the existence of UPR in GD derived skin fibroblasts. Wei et al. showed in one N370S/N370S Type 1 GD derived skin fibroblast line, a significant increase in mRNA level of the UPR related genes ATF6, BiP and Xbp1. This was accompanied by a concomitant increase in the protein level of active ATF6, BiP and phosphorylated eIF2α [17]. Mu et al. reported on UPR activation in cell lines that derived from GD patients homozygous for the N370S and the L444P mutations [24]. In another publication the efficacy of catechin in alleviating UPR in one GD derived line (N370S/N370S) has been documented [18].

In the present work we show UPR activation in skin fibroblasts derived from GD patients manifested by an increase in mRNA and protein levels of BiP and CHOP, splicing of Xbp1 and phosphorylation of eIF2α. We also show that in fibroblast lines derived from carriers of GD mutations there is UPR as well. Interestingly, the 84GG mutation is not expected to culminate in a mature protein [6]. Since there is UPR in carriers of this mutation, one has to assume that either the 84GG mutant RNA participates in translation of a very short peptide (26 amino acids long), shorter than the full size leader of GCase (38 amino acids long), thereby blocking ER entrance of newly synthesized proteins, or the 84GG mRNA-bound polyribosomes block ER entrance, thereby leading to development of ER stress and, as a result, to UPR. These hypotheses will have to be further tested.

ERAD and UPR are well conserved across species and *Drosophila* has become an important tool in studying these phenomena. The short life span of the fly with the sophisticated molecular and genetic tools for fast establishment of transgenic lines, and availability of deletions and mutations in any chosen gene have made it an attractive model, which allows fast screening and analyses of large populations. We could recapitulate UPR in two fly models: one corresponding to carriers of GD mutations and the other involving transgenic flies expressing the human N370S or the L444P mutant proteins.

All lysosomal enzymes are synthesized on ER bound polyribosomes and upon their entry into the ER undergo N-linked glycosylation and quality control, after which they shuttle to the Golgi apparatus. Following further modifications there, they are trafficked to the lysosomes. Therefore, all mutant lysosomal enzymes are expected to undergo ERAD and induce the UPR machinery. UPR has already been documented in other lysosomal diseases. Thus, in Fabry disease which results from mutation in the α-galactosidase-A encoding gene (α-Gal-A) and accumulation of the globotrioside Gb3, mutant variants undergo ERAD, which induces the UPR [57,58]. UPR has also been documented in skin fibroblasts from patients suffering from ceroid lupofusinosis (CLN) 1, 2, 3, 6 and 8, as well as in cells of patients with GM1 gangliosidosis (suffering from reduced activity of β-galactosidase), Tay Sachs disease (reduced activity of β-hexosaminidase A) and Niemann Pick type C2 (mutations in the NPC2 gene) [17]. Interestingly, in some model systems for lysosomal diseases UPR was not recapitulated. Farfel et al. [19] were unable to demonstrate UPR in neuronal cells that derived from knockout mice, lacking the *GBA* gene, or in animals or cells, treated with the GCase non-competitive inhibitor CBE. Lack of UPR in both of these cases is likely due to the absence of mutant GCase in the ER. Likewise, in NPC1-deficient mice and in an NPC1 cell-based model, created by knocking down the expression of NPC1 using RNA interference, there was no UPR [59]. Again, in both cases, no mutant protein was present in the ER to induce the UPR machinery.

In recent years association has been elegantly demonstrated between GD and PD, a neurodegenerative disease affecting 1% of individuals over 60 years old. Thus, there is a higher propensity among Type 1 GD patients and among carriers of GD mutations to develop PD in comparison to the non-GD population [31-55]. Brains of carriers of GD mutations who develop PD display Lewy bodies (LB) and loss of substantia nigra neurons [60]. Carriers of GBA mutations tend to have more cortical LBs than those of non-carriers (82% versus 43%, respectively) [61], suggesting that mutant GCase variants promote α-synuclein aggregation directly. Cullen et al. showed that expression of mutant human GCase, but not that of the normal counterpart, led to increase in α-synuclein accumulation in MES23.5, PC12, and HEK293 cell lines, arguing that mutant GCase has a direct role in α-synuclein accumulation, and most probably, aggregation [62].

Since carriers of GD mutations do not accumulate GlcCer in their brain and its build-up has not been demonstrated in brains of Type 1 GD patients, we raised the possibility that ERAD of mutant GCase contributes to the development of PD among GD patients and carriers of GD mutations. We have shown that parkin interacts with mutant GCase and mediates its Lysine 48 ubiquitination and proteasomal degradation [20]. We proposed that this interaction between parkin and mutant GCase leads to deleterious effect in dopaminergic cells caused by the accumulation of parkin substrates, which are potentially toxic. Our recent results (Bendikov-Bar and Horowitz, unpublished) show that mutant GCase competes with two known substrates of parkin, PARIS [63] and ARTS [64], whose accumulation in cells leads to apoptosis. PARIS is a transcription repressor of peroxisome proliferator-activated receptor gamma (PPARγ) coactivator-1α (PGC-1α) expression. PGC-1α is a master regulator of mitochondrial biogenesis [65]. Thus, PARIS accumulation impedes mitochondria biogenesis. Interestingly, PARIS accumulates in mouse models of parkin inactivation and in PD patients’ brains [63]. ARTS is a mitochondrial protein that initiates caspase activation, upstream of cytochrome c release in the mitochondrial apoptotic pathway [66].

We now extend our hypothesis for the role of mutant GCase in the development of PD. We propose that ERAD of mutant GCase leads to accumulation of parkin substrates, some of which are deleterious, like PARIS and ARTS. This accumulation leads to cell death. We also assume that UPR, as part of the cellular ER stress, induced by the persistent presence of mutant GCase in the ER, leads to cellular death. Therefore, both, ERAD of mutant GCase and UPR contribute to dopaminergic cell death and development of PD. It still remains to be tested whether and how mutant GCase leads to aggregation of α-synuclein.

In the present work we show that expression of the mutant fly orthologs of GBA or expression of human mutant N370S or L444P proteins leads to death at early stages of the fly development. The flies expressing mutant GCase in the dopaminergic/serotonergic cells develop locomotion dysfunction, reminiscent of PD. This is the first animal model in which carriers of GD mutations develop parkisonian signs.

In a recent publication, parkin insolubility was associated with lack of degradation of ubiquitinated proteins and accumulation of α-synuclein and parkin in autophagosomes, suggesting autophagic defects in PD [67]. To test parkin’s role in mediating autophagic clearance, the authors used lentiviral gene transfer to express human wild type or mutant parkin (T240R) with α-synuclein in the rat striatum. Lentiviral expression of α-synuclein led to accumulation of autophagic vacuoles, while co-expression of parkin with α-synuclein facilitated autophagic clearance. Expression of parkin loss-of-function mutation did not affect autophagic clearance. Taken together, the data suggested that functional parkin regulates autophagosome clearance. It is possible, and remains to be proven, that the interaction between parkin and mutant GCase variants in dopaminergic cells attenuates normal autophagy, which leads to α-synuclein aggregation.

Our results strongly indicate a direct association between mutant GCase and development of Parkinsonian signs in the fly. However, there is another paradigm, arguing that insufficient lysosomal mutant GCase activity leads to substrate accumulation (GlcCer or glucosylsphingosine), α-synuclein aggregation, block in trafficking of GCase to lysosomes and development of PD [60,62,68-70]. Thus, Sardi et al. showed that in brain sections derived from 12 months old D409V homozygous mice (but not from D409V heterozygous animals) there are α-synuclein and ubiquitin aggregates in the hippocampus, cerebral cortex and cerebellum. Memory deficits were detected in these mice at 6 months of age [68]. Administration of normal enzyme to the brain using gene therapy with an AAV derived vector, expressing a normal human GBA cDNA, significantly reduced the aggregation of ubiquitin and α-synuclein and ameliorated the memory deficit [71].

Development of PD in carriers of GD mutations implies that the presence of a mutant GBA allele is a dominant predisposing factor. This is a unique case of an autosomal recessive metabolic disease with a dominant element, namely the tendency of carriers of GD mutations to develop PD.

Dominance results either from haploinsufficiency or from gain of function. If haploinsufficiency accounts for the development of PD in carriers of GD mutations, it implies insufficient GCase activity in the dopaminergic neurons. Why is it not manifested in macrophages, in which case the disease would have been dominant? If, alternatively, the dominance results from gain of function, then its development depends on accumulation of enough deleterious product (mutant GCase, in our case), as in the case of Alzheimer disease, which displays age dependent accumulation of β-amyloid and tau, or Huntington disease, which exhibits accumulation of huntingtin [72-74]. Our results suggest the gain of function alternative.

## Conclusions

In this study we show that UPR is activated in GD patients as well as in carriers of GD mutations. In *Drosophila* models for carriers of different GD mutations, UPR is activated as well, and locomotion deficits are observed in the aging flies as a result of the presence of mutant GCase.

## Consent

Written informed consent was obtained from the patients for publication of this paper.

## Abbreviations

GD: Gaucher disease; GCase: Glucocerebrosidase; ER: Endoplasmic reticulum; ERQC: ER quality control; ERAD: ER associated degradation; UPR: Unfolded protein response; ATF6: Activating transcription factor 6; GlcCer: Glucosylceramide; eIF2α: Eukaryotic Initiation Factor 2α; CBE: Conduritol-B-epoxide; PD: Parkinson disease; LB: Lewy body.

## Competing interests

The authors declare no competing interests.

## Authors’ contributions

GM designed the experiments, performed them and wrote the manuscript; SRL constructed the *Drosophila* expressing plasmids: MF supplied most human cell lines; HS supplied *Drosophila* materials and participated in manuscript preparation; DS participated in designing of experiments and in preparation of the manuscript; MH designed the experiments and participated in the manuscript preparation. All authors read and approved the final manuscript.

## Supplementary Material

Additional file 1: Figure S1 CBE treatment does not induce UPR. A. To test the effect of substrate accumulation on induction of the UPR machinery, normal skin fibroblasts were treated with 200 mM of the GCase non-competitive inhibitor, CBE (Sigma-Aldrich, Israel) for 10 days. Enzymatic activity was tested after a day and 10 days of treatment, using 4-MUG as a substrate. B. Following 10 days of CBE treatment RNA was prepared from the cells and activation of UPR was tested by following CHOP and BiP mRNA, using quantitative RT-PCR with primers specific for human BiP (Human-GRP78-RT-F and Human-GRP78-RT-R, Table 1) or CHOP (Human-CHOP-RT-F and Human-CHOP-RT-R, Table 1). GAPDH was used as a normalizing control (amplified with primers: Human-GAPDH-RT-F and Human-GAPDH-RT-R, Table 1). As control for UPR activation, cells were treated for 3 hours with 150 nM of Thapsigargin (Sigma Aldrich, Israel), after which RNA was prepared and used for quantitative RT-PCR. Significance: * < 0.05; ** < 0.01. Dark box: CHOP; Light box: BiP. Click here for file
