# Supplementary figures and images for: Unfolded protein response in Gaucher disease: from human to Drosophila
Source: Orphanet J Rare Dis. 2013 Sep 11;8:140. doi: 10.1186/1750-1172-8-140 (PMC3819655; doi:10.1186/1750-1172-8-140)

Supplementary Figure 1.

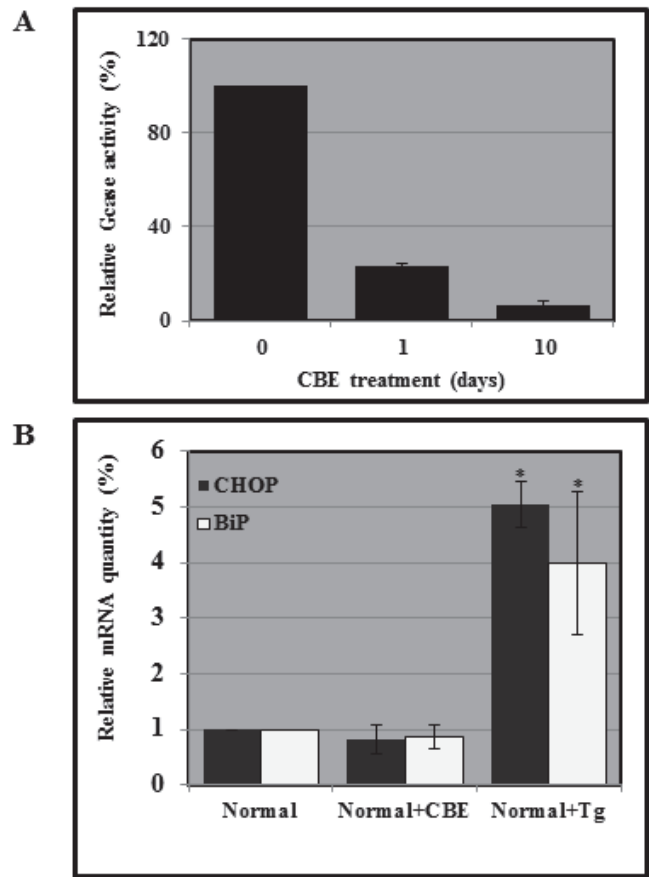

Supplement: Additional file 1: Figure S1 — CBE treatment does not induce UPR. A. To test the effect of substrate accumulation on induction of the UPR machinery, normal skin fibroblasts were treated with 200 mM of the GCase non-competitive inhibitor, CBE (Sigma-Aldrich, Israel) for 10 days. Enzymatic activity was tested after a day and 10 days of treatment, using 4-MUG as a substrate. B. Following 10 days of CBE treatment RNA was prepared from the cells and activation of UPR was tested by following CHOP and BiP mRNA, using quantitative RT-PCR with primers specific for human BiP (Human-GRP78-RT-F and Human-GRP78-RT-R, Table 1) or CHOP (Human-CHOP-RT-F and Human-CHOP-RT-R, Table 1). GAPDH was used as a normalizing control (amplified with primers: Human-GAPDH-RT-F and Human-GAPDH-RT-R, Table 1). As control for UPR activation, cells were treated for 3 hours with 150 nM of Thapsigargin (Sigma Aldrich, Israel), after which RNA was prepared and used for quantitative RT-PCR. Significance: * < 0.05; ** < 0.01. Dark box: CHOP; Light box: BiP. [file 1750-1172-8-140-S1.pdf]
